# Supplementary material for: Structural basis for inhibition of the Cation-chloride cotransporter NKCC1 by the diuretic drug bumetanide
Source: Nat Commun. 2022 May 18;13:2747. doi: 10.1038/s41467-022-30407-3 (PMC9117670; doi:10.1038/s41467-022-30407-3)
Supplement: Supplementary file 1 — Supplementary Information [file 41467_2022_30407_MOESM1_ESM.pdf]

Supplementary Information

Structural Basis for Inhibition of the Cation-chloride Cotransporter NKCC1 by the Diuretic Drug Bumetanide

Yongxiang Zhao *et al*.

Supplementary Table 1 | Statistics of cryo-EM data acquisition and processing and model building

**Data collection/Processing**			
	NKCC1$_{iii}$/ bumetanide	NKCC1$_{iii}$/ apo	NKCC1$_{ii}$ bumetanide
Microscope	Titan Krios K3 camera	Titan Krios K3 camera	Titan Krios K3 camera
Voltage (kV)	300	300	300
Defocus range (μm)	-0.7 – -3.5	-0.7 – -3.5	-0.7 – -3.5
Pixel size (Å)	1.06	1.06	1.06
Total electron dose ($e^-/Å^2$)	47	47	47
Exposure time (s)	2.5	2.5	2.5
Number of movies	3,888	4,289	4,266
Number of frames per movie	40	40	40
Initial particle number	504,481	536,763	1,033,074
Final particle number	39,237	75,001	37,297
Resolution (unmasked, Å)	3.7	3.9	4.1
Resolution (masked, Å)	2.9	3.3	3.6
**Refinement and Validation**			
Number of atoms	12658	6,416	6,580
R.M.S deviation			
Bond length (Å)	0.005	0.003	0.004
Bond angles (°)	0.812	0.702	0.728
**Ramachandran**			
Favored (%)	90.12%	90.64%	92.58%
Allowed (%)	9.88%	9.36%	7.2%
Outlier (%)	0.00%	0.00%	0.21%
Molprobity score	2.30	2.11	2.06

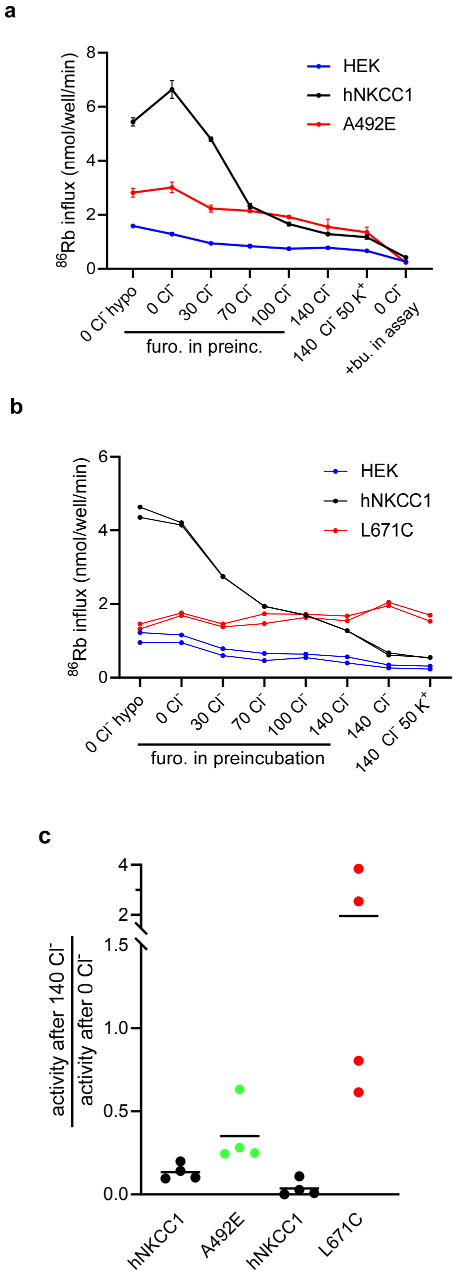

Supplementary Fig. 1 Constitutive activity of human NKCC1 A492E and L671C mutants in HEK293 cells. Human NKCC1 activity was assayed by a 1-minute ^{86}Rb influx after 1-hour preincubation in media with various Cl$^-$ concentrations: lowered extracellular [Cl$^-$] in turn reduces intracellular [Cl$^-$] and this is known to be required for phosphorylation and activation of NKCC1; elevated [Cl$^-$] conversely leads to dephosphorylation and inactivation of NKCC1. a-b) Mutations A492E (a) and L671C (b) exhibit significant activity even after preincubation in high [Cl$^-$], indicting significant activity of these mutants without phosphorylation. Individual experiments are shown in (a) and (b): n=3 determinations with SEM and mean in (a), duplicate determinations in (b). c) Summary data showing the ratio of activities following preincubation in 140 mM Cl$^-$ compared to preincubation in 0 mM Cl$^-$ (n=4 experiments each: control with A492E and control with L671C; lines indicate means).

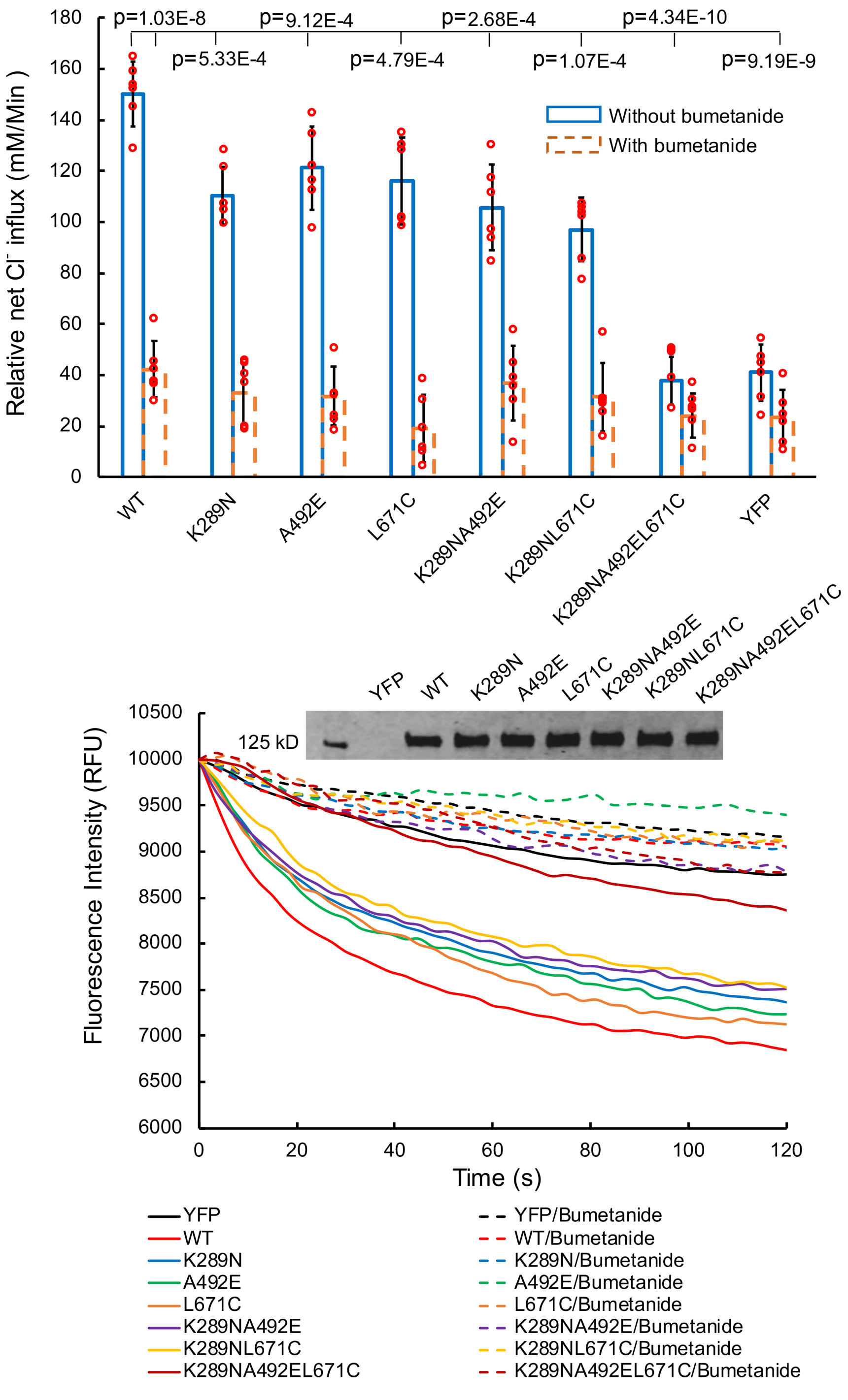

Supplementary Fig. 2 Characterization of NKCC1 mutants in insect cells. Top, Cl^- transport rates were measured for wildtype human NKCC1 and mutants relevant for cryo-EM studies. Each circle represents one kinetic measurement of a single sample in a 96 well plate. Unpaired one-tailed Student's t-tests are used for statistical analyses (n=6; data are presented as mean values +/- SD). Bottom, averaged raw traces show that NKCC1-mediated Cl^- influx leads to quenching of YFP fluorescence. Note, all constructs, except for the $NKCC1_{iii}$ construct (K289N/A492E/L671C), show bumetanide-sensitive transport activity. All NKCC1 constructs show similar expression level in insect cells as indicated by western blot.

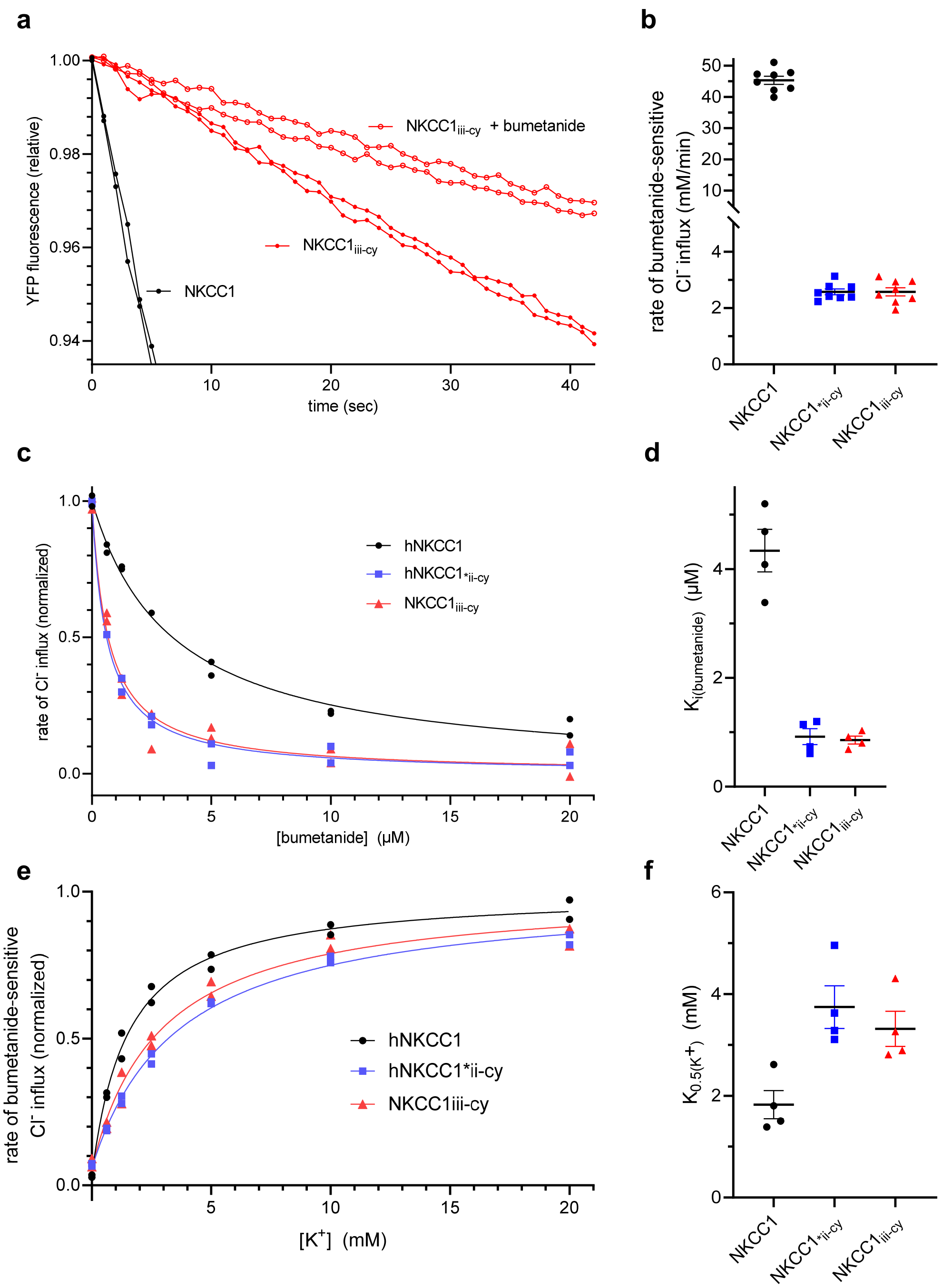
a
b
c
d
e
f
YFP fluorescence (relative)
1.00
0.98
0.96
0.94
time (sec)
NKCC1iii-cy + bumetanide
NKCC1iii-cy
NKCC1
rate of bumetanide-sensitive Cl- influx (mM/min)
NKCC1
NKCC1*ii-cy
NKCC1iii-cy
rate of Cl- influx (normalized)
[bumetanide] (μM)
hNKCC1
hNKCC1*ii-cy
NKCC1iii-cy
Ki(bumetanide) (μM)
rate of bumetanide-sensitive Cl- influx (normalized)
[K+] (mM)
K0.5(K+) (mM)

Supplementary Fig. 3 Functional examination of NKCC1$_{iii}$ in HEK293 cells. NKCC1$_{iii}$ and NKCC1$_{*ii}$ (A492E/L671C) tagged with CFP and YFP in the C-terminus were studied in HEK293 cells; it will be noted that in these experiments NKCC1$_{iii}$ and NKCC1$_{*ii}$ were functionally indistinguishable. For experiments designed to measure Cl^- fluxes these cells were also transfected with a soluble Cl^--sensing YFP; the amount of soluble Cl^--sensing YFP was more than 10 times that of the membrane-expressed FRET constructs. a) YFP fluorescence traces upon introduction of regular medium following a 2-hour pre-incubation in Cl^--free medium. Results with only NKCC1 and NKCC1$_{iii\text{-}cy}$ (+/- bumetanide) are shown for simplicity: these are 6 of the 48 traces analyzed for panel (c). b) Rate of bumetanide-sensitive Cl^- influx in regular medium in the eight experiments examined in panels (c-f). c,d) Inhibition of Cl^- influx by bumetanide in NKCC1, NKCC1$_{*ii\text{-}cy}$ and NKCC1$_{iii\text{-}cy}$. Bumetanide at indicated concentrations was included in regular medium for the 0.7-minute flux period. For these experiments, fluxes were analyzed between 0.5-0.7 minutes after medium addition to allow a 0.5-minute period for bumetanide binding, and duplicate determinations at each point in one experiment are shown in (c). d) K_i values for bumetanide inhibition from least-squares fits to the data in four experiments, including the experiment shown in panel (c). Note that these K_i values are not directly comparable to previously reported $K_{i(bumetanide)}$ values which were carried out with bumetanide pre-incubation at a reduced $[Cl^-]$[43]. e,f) Dependence of bumetanide-sensitive Cl^- influx on K^+ concentration. e) Results of one experiment in which $[K^+]$ was varied (replaced by Na^+); points indicate the rate of bumetanide-sensitive Cl^- influx with duplicate determinations shown. f) $K_{0.5}$ values for K^+ stimulation from least-squares fits in four experiments including the experiment shown in panel (e); the $K_{0.5}$ values for NKCC1$_{*ii\text{-}cy}$ and NKCC1$_{iii\text{-}cy}$ are different from that of NKCC1 by unpaired two-sided Student's t-test (p=0.009, p=0.015, respectively). Panels b,d,e represent data from n=8,4,4 experiments respectively, with lines and error bars representing mean values +/- SEM.

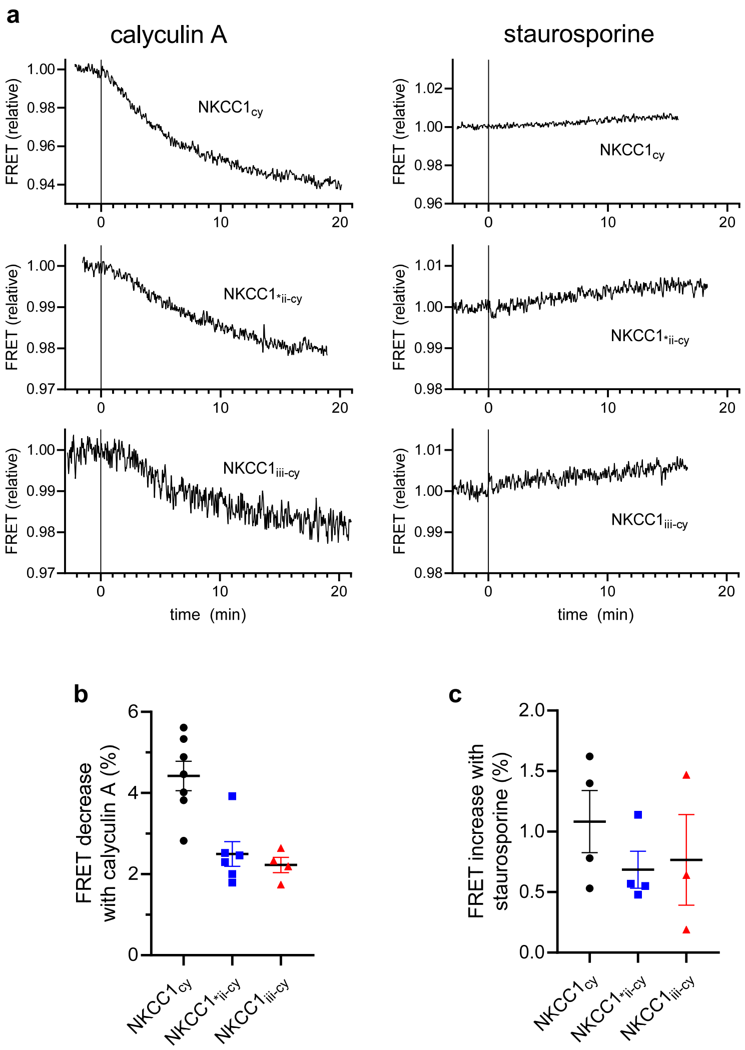

Supplementary Fig. 4 Regulatory change in the C-terminus of NKCC1 indicated by FRET. NKCC1 constructs (NKCC1$_{cy}$, NKCC1$_{iii\text{-}cy}$ NKCC1$_{*ii\text{-}cy}$) tagged with CFP and YFP in the C-terminus were expressed in HEK293 cells, and FRET$_{norm}$ was measured in cells plated on coverslips as in reference[57]. a) Representative traces of FRET following addition at t=0 of calyculin A (250 nM) to cause phosphorylation of NKCC1 (left panels); the decrease in FRET in human NKCC1$_{cy}$ is similar to but smaller than the 11% decrease previously found for a similar shark NKCC1 construct[57]. Also shown is the effect of staurosporine (4 µM) to block NKCC1 phosphorylation (right panels); we found a small increase in FRET suggesting that there is some constitutive activation of the C-terminus change in regular medium. b,c) Summary of the magnitude of FRET changes with calyculin and staurosporine in five experiments ((b) n=7,7,4 traces respectively for the three constructs, (c) n=4,4,3 traces respectively; lines and error bars represent mean values +/- SEM). In panel (b) the fractional decreases for NKCC1$_{*ii\text{-}cy}$ and NKCC1$_{iii\text{-}cy}$ are significantly smaller than for NKCC1$_{cy}$ by unpaired two-sided Student's t-test (p=0.002 in each case); in panel (c) there are no significant differences among constructs in the magnitude of the staurosporine-induced increase in FRET.

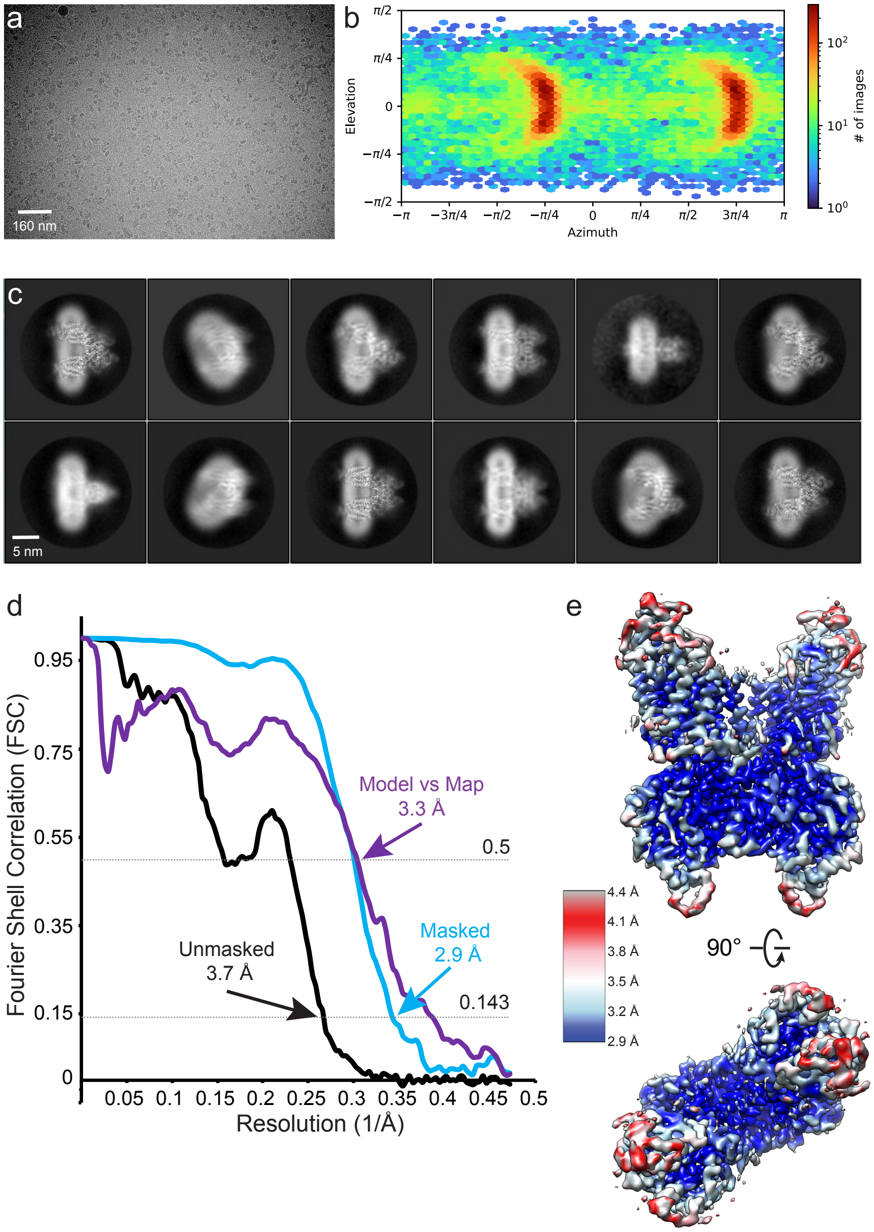

Supplementary Fig. 5 Cryo-EM structure of the human NKCC1$_{iii}$ construct bound with bumetanide. a) A representative micrograph of NKCC1$_{iii}$/bumetanide recorded with a titan Krios microscope. b) Angular distribution plot of all particle projections as output by cryoSPARC 3.0. c) 2D class averages of NKCC1$_{iii}$/bumetanide showed well-resolved structural features for both the transmembrane and cytosolic domains. d) Gold-standard FSC curves calculated after cryoSPARC 3.0 non-uniform refinement. e) Local resolutions calculated in cryoSPARC 3.0.

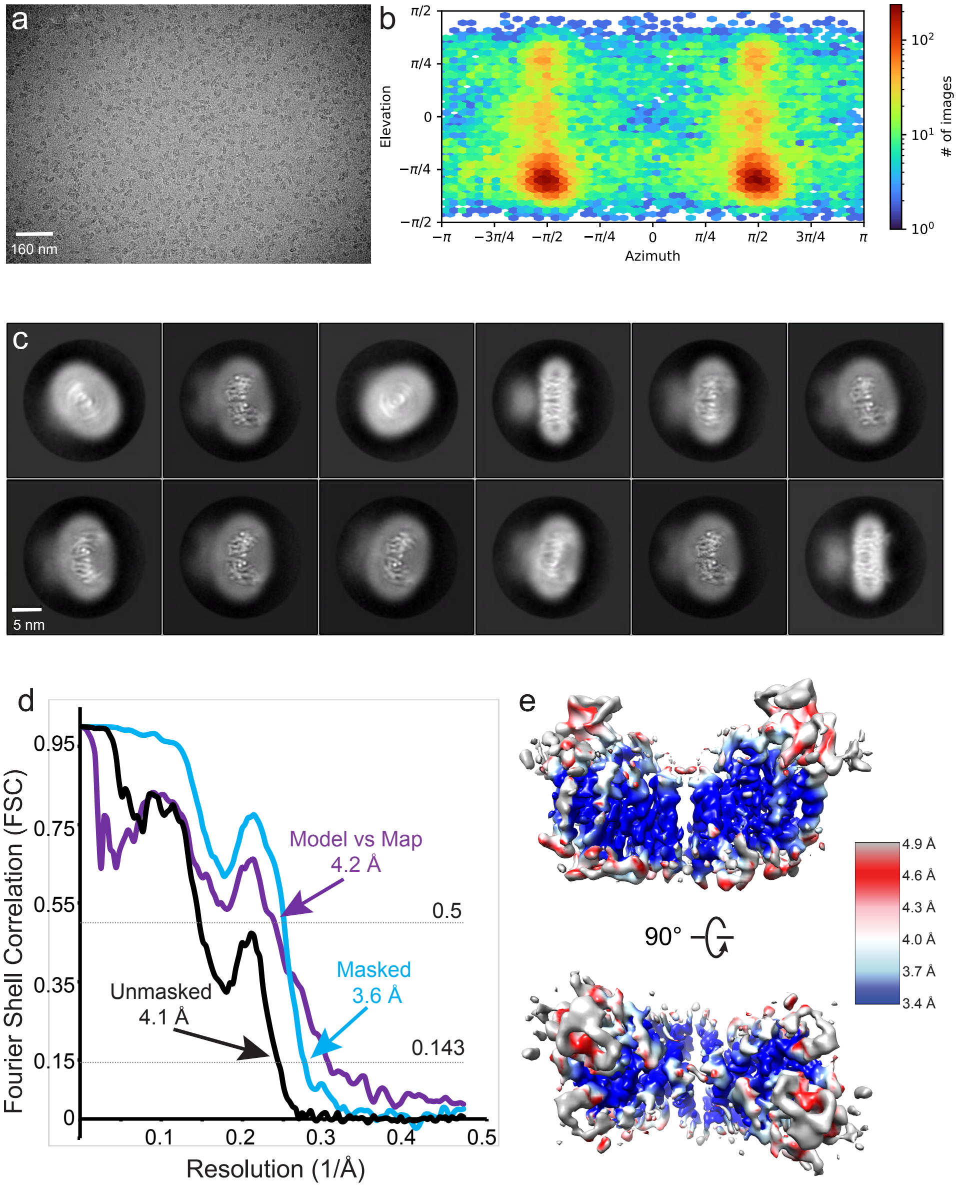

Supplementary Fig. 6 Cryo-EM structure of the human NKCC1$_{ii}$ construct bound with bumetanide. a) A representative micrograph of NKCC1$_{ii}$/bumetanide recorded with a titan Krios microscope. b) Angular distribution plot of all particle projections as output by cryoSPARC 3.0. c) 2D class averages of NKCC1$_{ii}$/bumetanide showed well-resolved structural features for the transmembrane, but not the cytosolic domain. d) Gold-standard FSC curves calculated after cryoSPARC 3.0 non-uniform refinement. e) Local resolutions calculated in cryoSPARC 3.0.

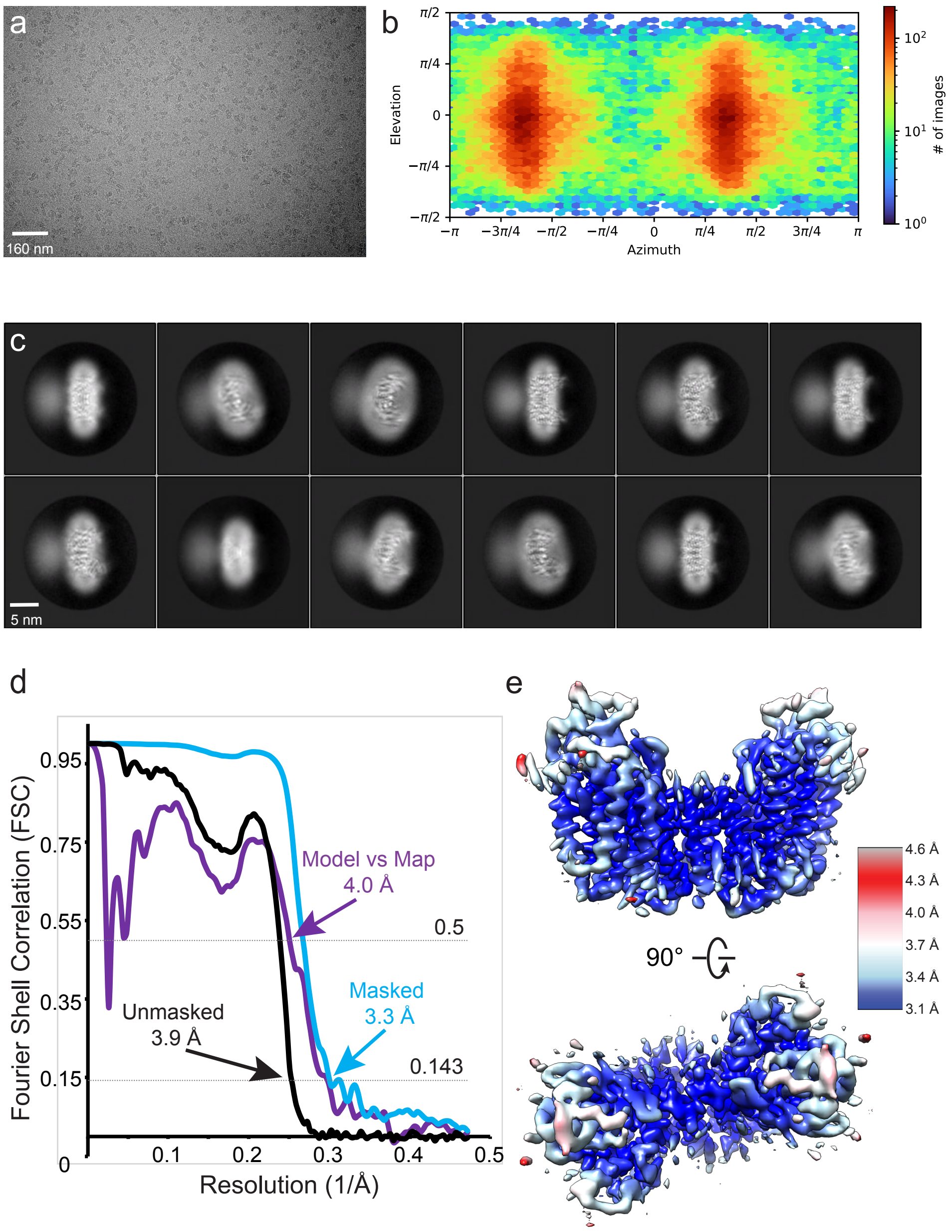

Supplementary Fig. 7 Cryo-EM structure of the human NKCC1$_{iii}$ construct without bumetanide (apo). a) A representative micrograph of NKCC1$_{iii}$/apo recorded with a titan Krios microscope. b) Angular distribution plot of all particle projections as output by cryoSPARC 3.0. c) 2D class averages of NKCC1$_{iii}$ showed well-resolved structural features for the transmembrane, but not the cytosolic domain. d) Gold-standard FSC curves calculated after cryoSPARC 3.0 non-uniform refinement. e) Local resolutions calculated in cryoSPARC 3.0.

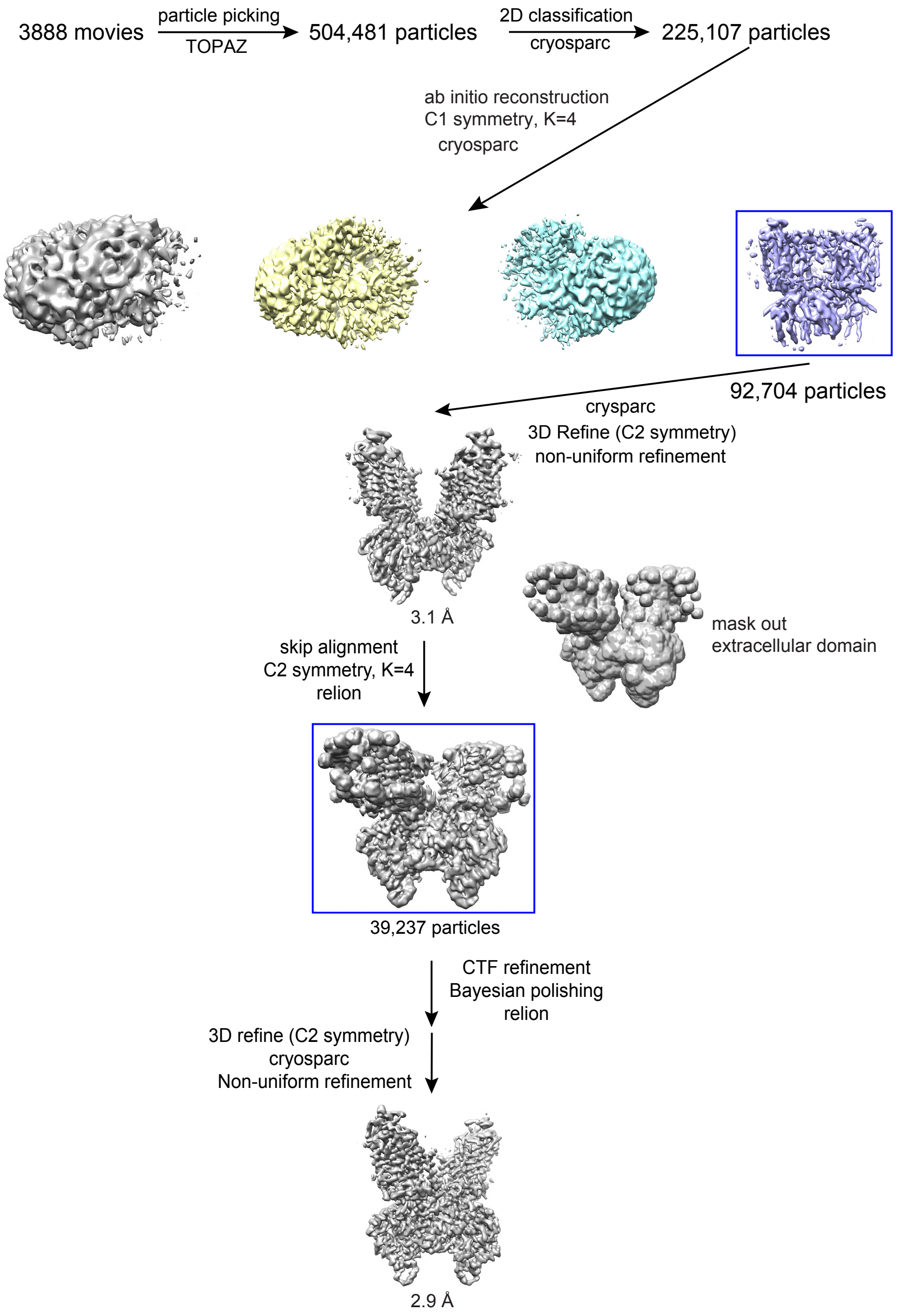

Supplementary Fig. 8 Reconstruction of the human NKCC1$_{iii}$ construct bound with bumetanide. Flow chart of image processing for the NKCC1$_{iii}$/bumetanide dataset.

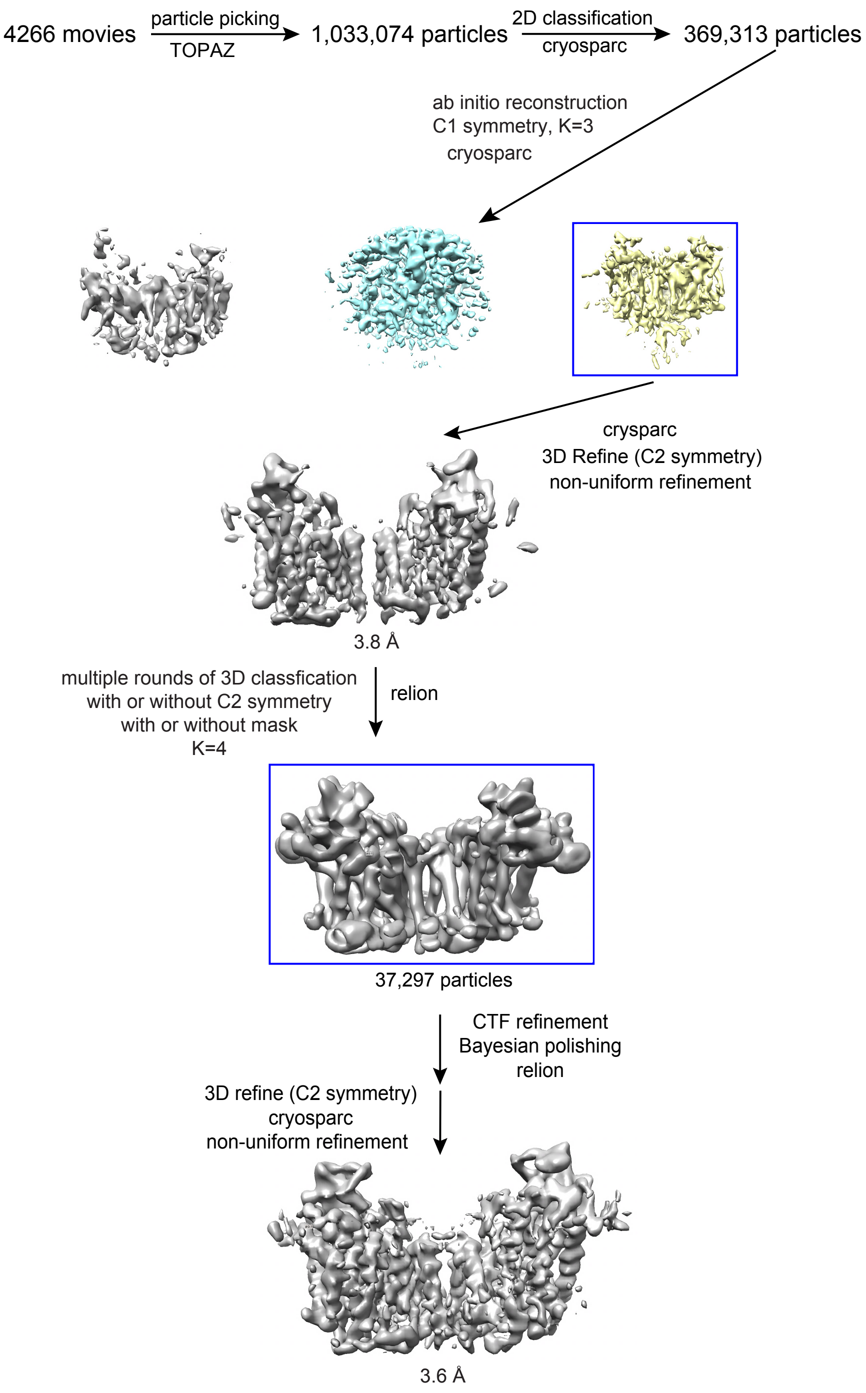

Supplementary Fig. 9 Reconstruction of the human NKCC1$_{ii}$ construct bound with bumetanide. Flow chart of image processing for the NKCC1$_{ii}$/bumetanide dataset.

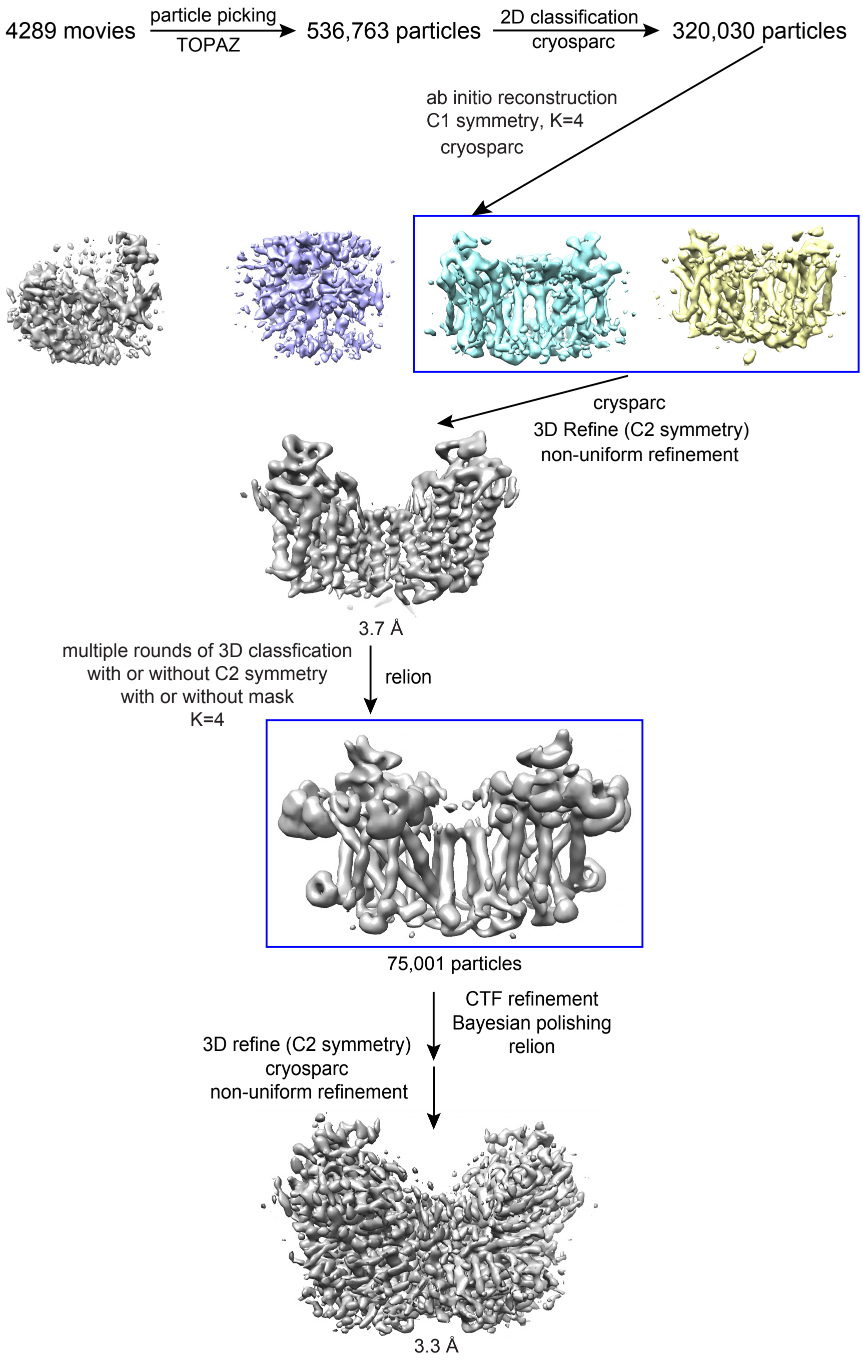

Supplementary Fig. 10 Reconstruction of the human NKCC1$_{iii}$ construct. Flow chart of image processing for the NKCC1$_{iii}$/apo dataset.

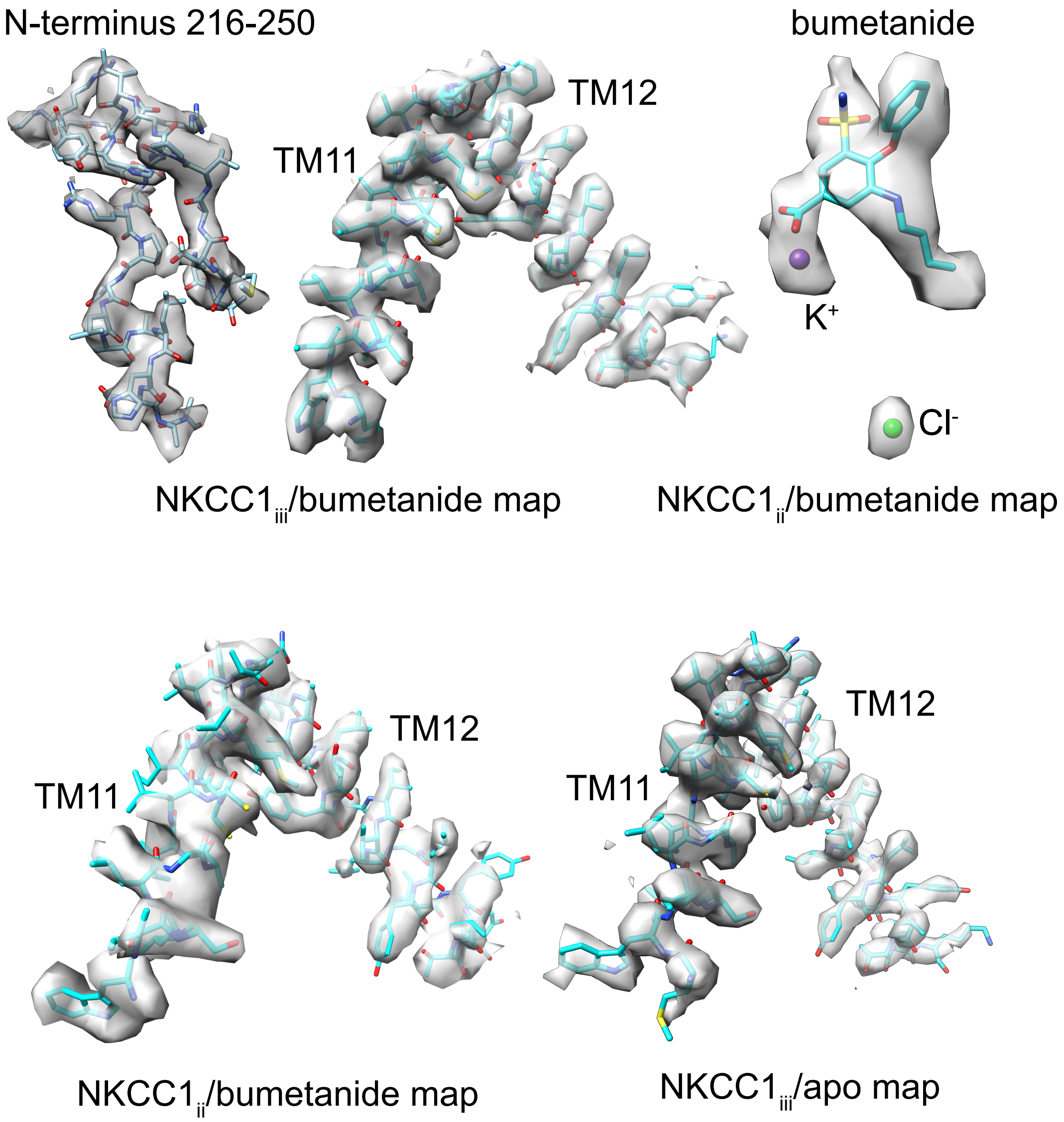

Supplementary Fig. 11 EM density maps of representative regions from three NKCC1 structures. The final models shown in stick are docked into experimental densities.

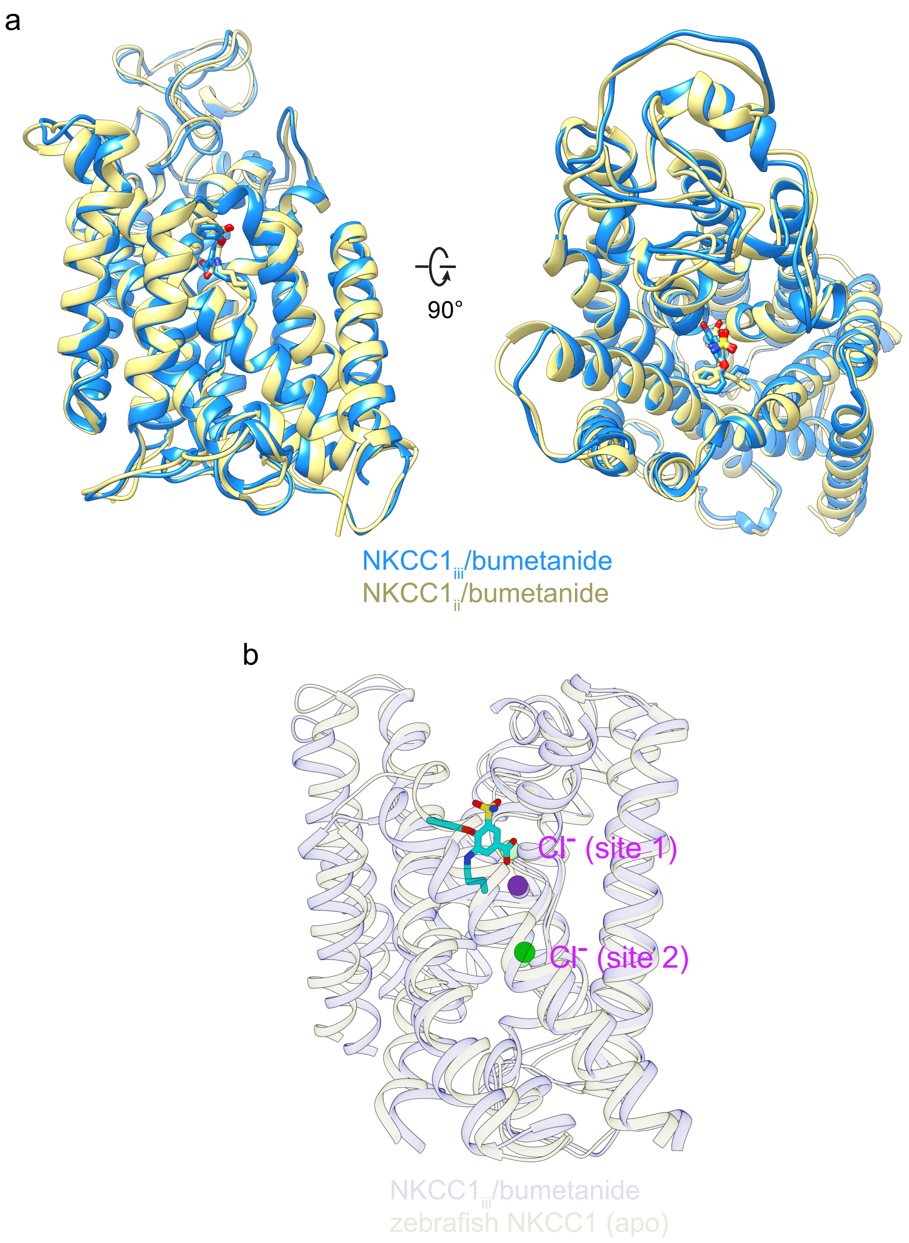

Supplementary Fig. 12 Comparison of the NKCC1$_{iii}$/bumetanide and NKCC1$_{ii}$/bumetanide structures. a) Bumetanide adopts a similar pose in both structures and arrests NKCC1 in an almost identical outward-open conformation. b) Superimposition of transmembrane domains of zebrafish NKCC1 (apo) and human NKCC1/bumetanide structures shows that the Cl^- in site 1 clashes with the carboxyl group of bumetanide (stick; cyan). Cl^- ions (green) and K^+ (purple) are shown as spheres. Ions identified in zebrafish NKCC1 are rendered semi-transparent for distinction.

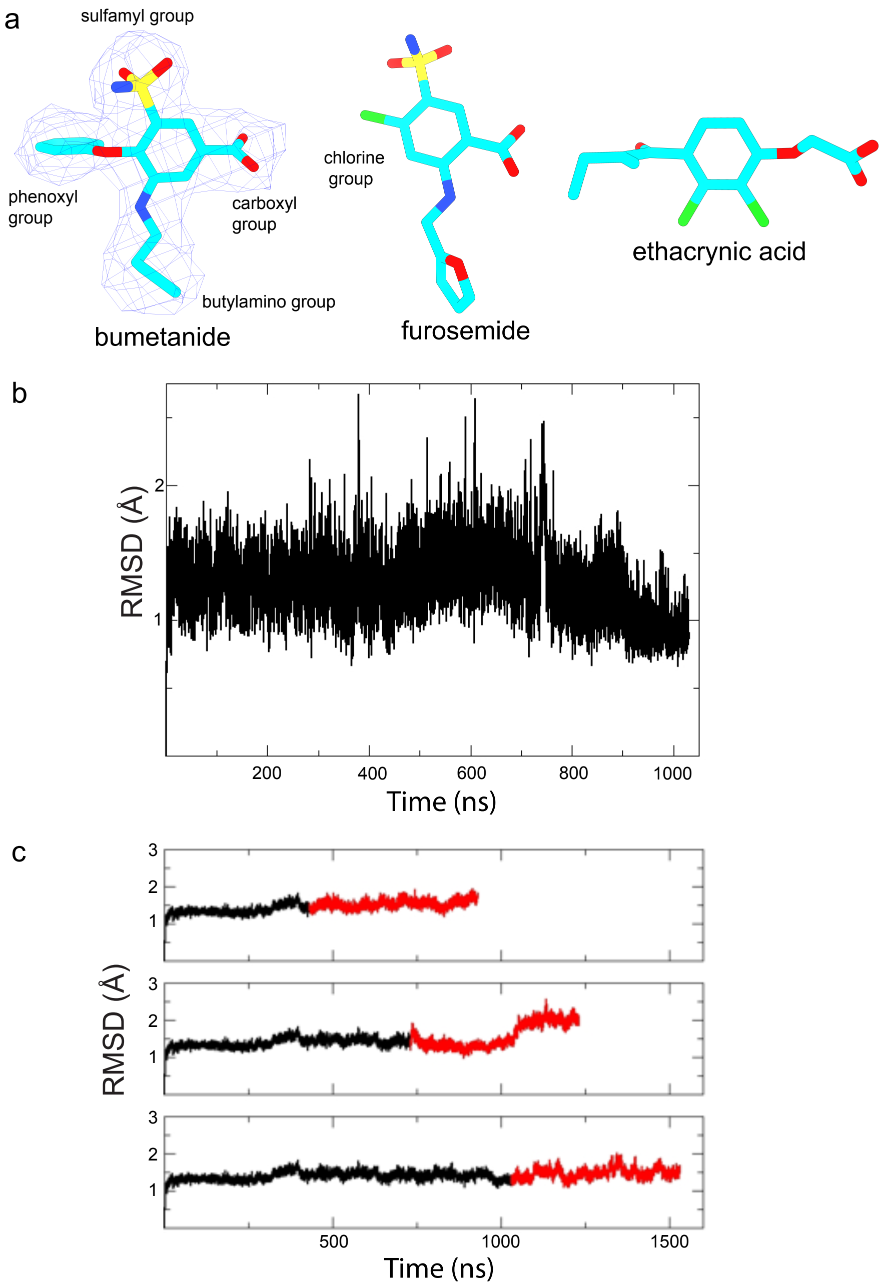

Supplementary Fig. 13 Bumetanide binding site. a) Bumetanide assumes a cross-shaped configuration. Chemical structures of furosemide and ethacrynic acid are shown for comparison. The blue mesh represents experimental cryo-EM density. b) Root mean square deviation (RMSD) of bumetanide with respect to the experimental pose during MD simulations. The RMSD was computed after aligning the backbone of the transmembrane helices. c) Structures of NKCC1$_{iii}$/bumetanide at 400 ns, 700 ns, and 1000 ns simulation were edited to remove bumetanide; solvent as allowed to fill the free space left by removal of bumetanide, after which each configuration was simulated for further 500 ns. Red lines: the RMSD with respect to the experimental structure of the transmembrane core (backbone atoms) after the removal of bumetanide. Black lines: shown for comparison, the RMSD of the transmembrane core before the removal of bumetanide.

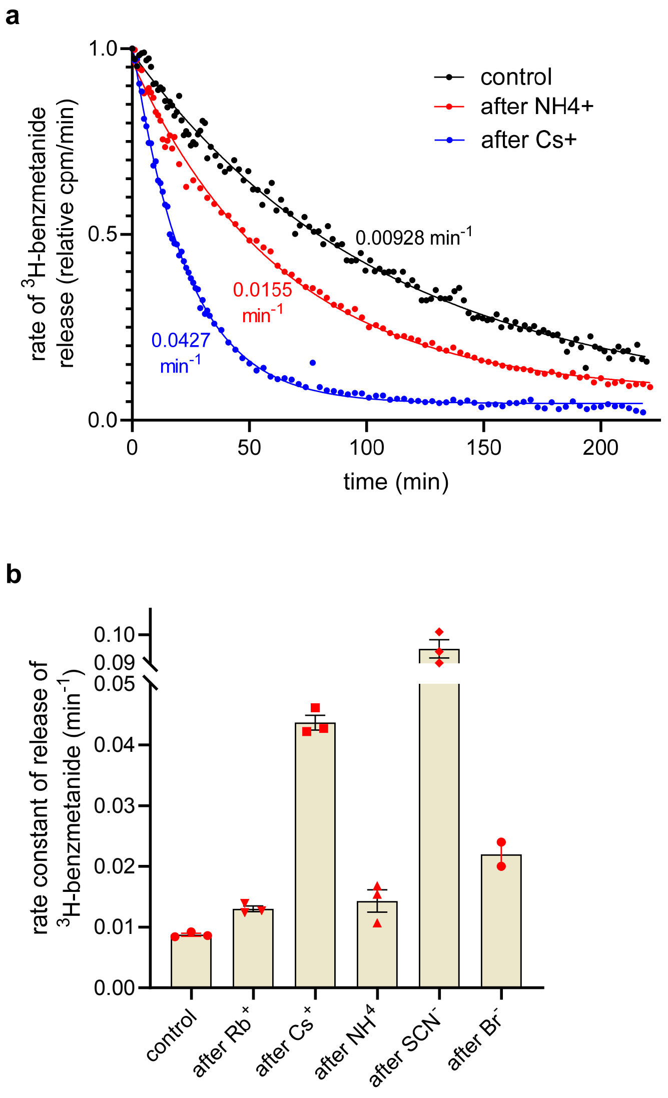

Supplementary Fig. 14 The rate of ^{3}H-benzmetanide release from shark NKCC1 depends upon co-occluded ions. ^{3}H-benzmetanide was bound to NKCC1 in an intact shark rectal gland during a 1-minute perfusion period in the presence of various ions: 270 mM Na^+ in all cases; 5 mM K^+, Cs^+, or NH_4^+; and 270 mM Cl^-, SCN^-, or Br^-. After 5 minutes for washout of non-specifically bound ^{3}H-benzmetanide, dissociation into shark Ringer's solution was monitored at 0.5 - 3 minutes time intervals. Note that the effect of ions present during ^{3}H-benzmetanide binding is "remembered" for several hours at 15°C, demonstrating occlusion of the ions with the bound ^{3}H-benzmetanide. a) Representative examples of the release of ^{3}H-benzmetanide following binding either in control shark Ringer's solution (black), or with K^+ substituted by NH_4^+ (red), or by Cs^+ (blue). Note that ion substitutions are only for a 3-minute period during which ^{3}H-benzmetanide exposure occurs. Dots represent the normalized raw data of ^{3}H-benzmetanide in each sample, thin lines are best fit of single exponential decays (rate constants shown in the Figure). b) Summary data for rate constants of ^{3}H-benzmetanide dissociation after binding with various ions (n=3 experiments except n=2 for Br^-, including those shown in (a); bars and error bars represent mean values +/- SEM).

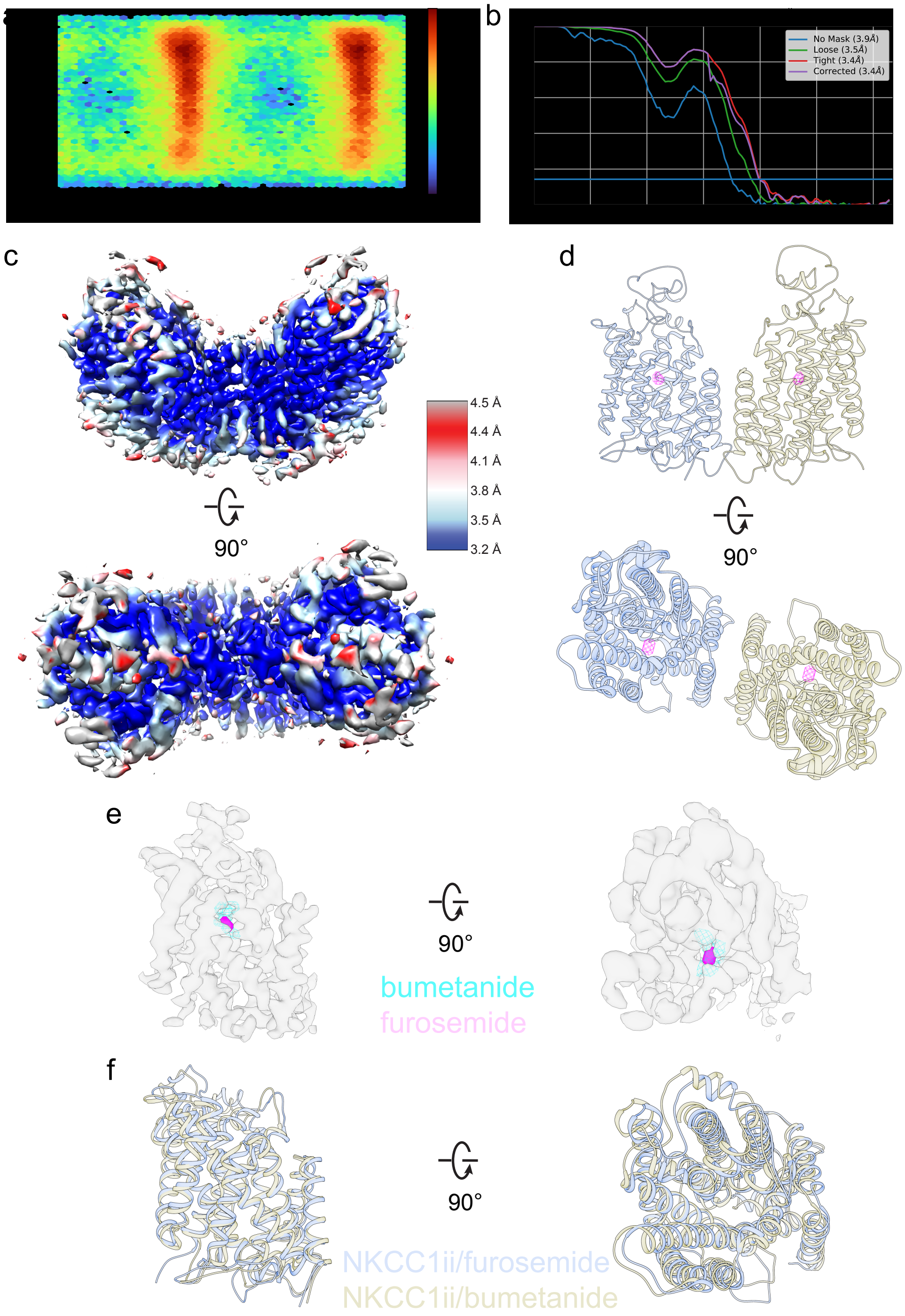

Supplementary Fig. 15 Cryo-EM structure of the human NKCC1$_{ii}$ construct bound with furosemide. a) Angular distribution plot of all particle projections as output by cryoSPARC 3.0. b) Gold-standard FSC curves calculated after cryoSPARC 3.0 non-uniform refinement. c) Local resolutions calculated in cryoSPARC 3.0. d) Furosemide densities (magenta meshes) is shown with NKCC1 structure (ribbon). e) Furosemide (magenta) and bumetanide (cyan mesh) occupy the same general loop diuretics packet. f) NKCC1$_{ii}$ adopts a similar outward-open state when bou nd with furosemide or bumetanide.

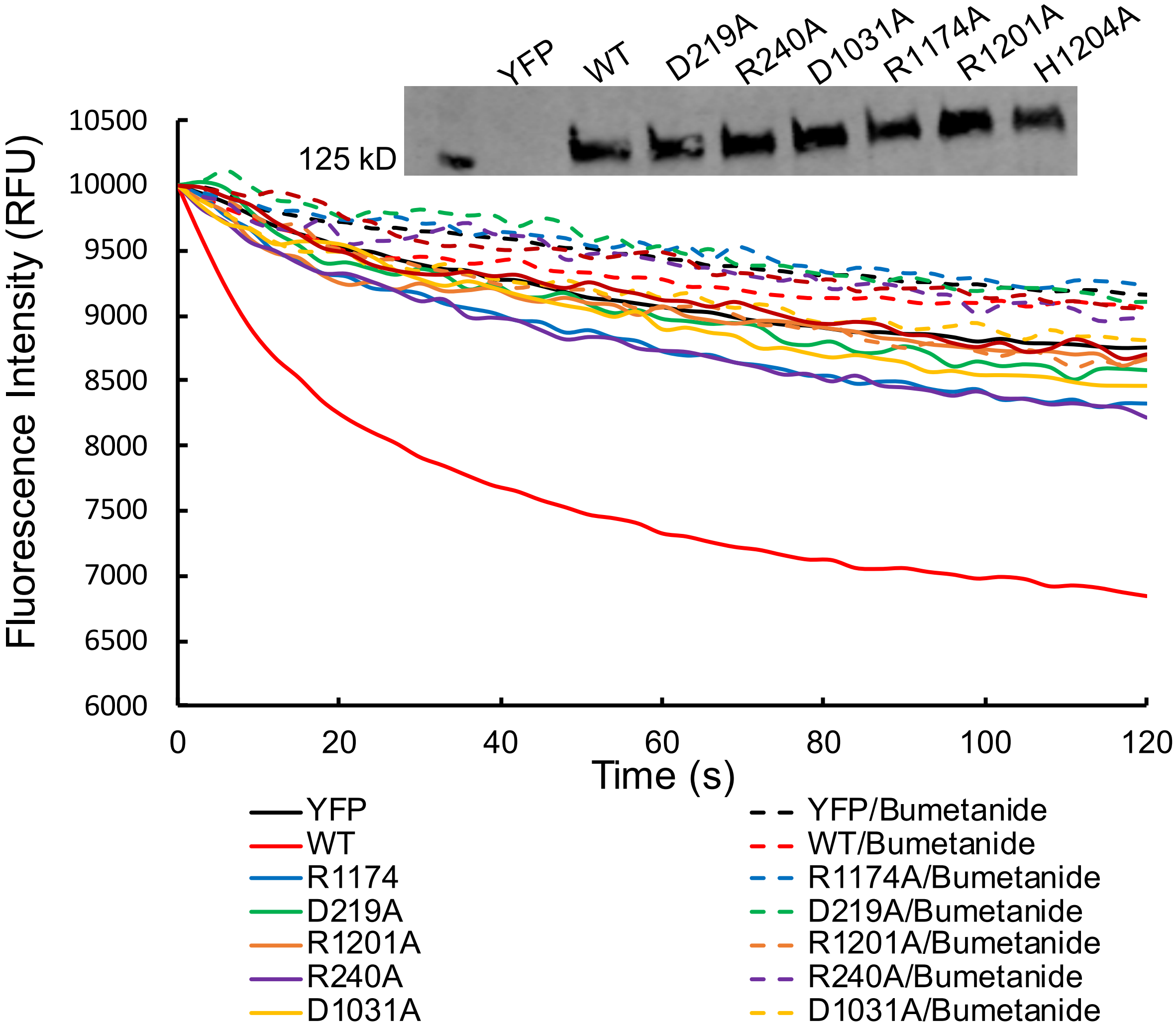

Supplementary Fig. 16 Functional analyses of NKCC1 mutants that affect association between the N- and C-terminal domains. NKCC1 mutants show reduced transport activity due to disrupted association between the N- and C-terminal domains. All mutants show normal expression as the wildtype NKCC1 transporter in western blot assay.

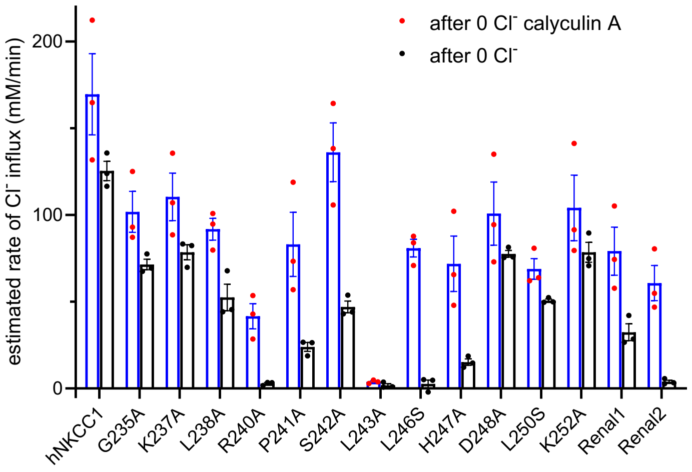

Supplementary Fig. 17 Activity of human NKCC1 with mutation in residues 235-252 in the N-terminus. Human NKCC1 activity was measured in HEK293 cells during a 0.5-minute incubation in regular medium following 90-minutes preincubation in a 140 mM *N*-Methyl-D-glucamine (NMDG) gluconate medium (0 Cl^-) either with (red points) or without (black points) addition of calyculin A for the final 15 minutes of preincubation. Calyculin A inhibits PP1 and usually assures full phosphorylation of NKCC1. Mutations are indicated in the figure; the "Renal1" mutation substitutes the NKCC2 sequence PKVNRPSLLEIHEQ for the equivalent NKCC1 sequence ^{236}EKLLRPSLAELHDE^{249}, and "Renal2" additionally includes S242A. Rates of NKCC1-mediated Cl^- influx were measured from the slope of the YFP fluorescence change (n=3 experiments; bars and error bars represent mean values +/- SEM).

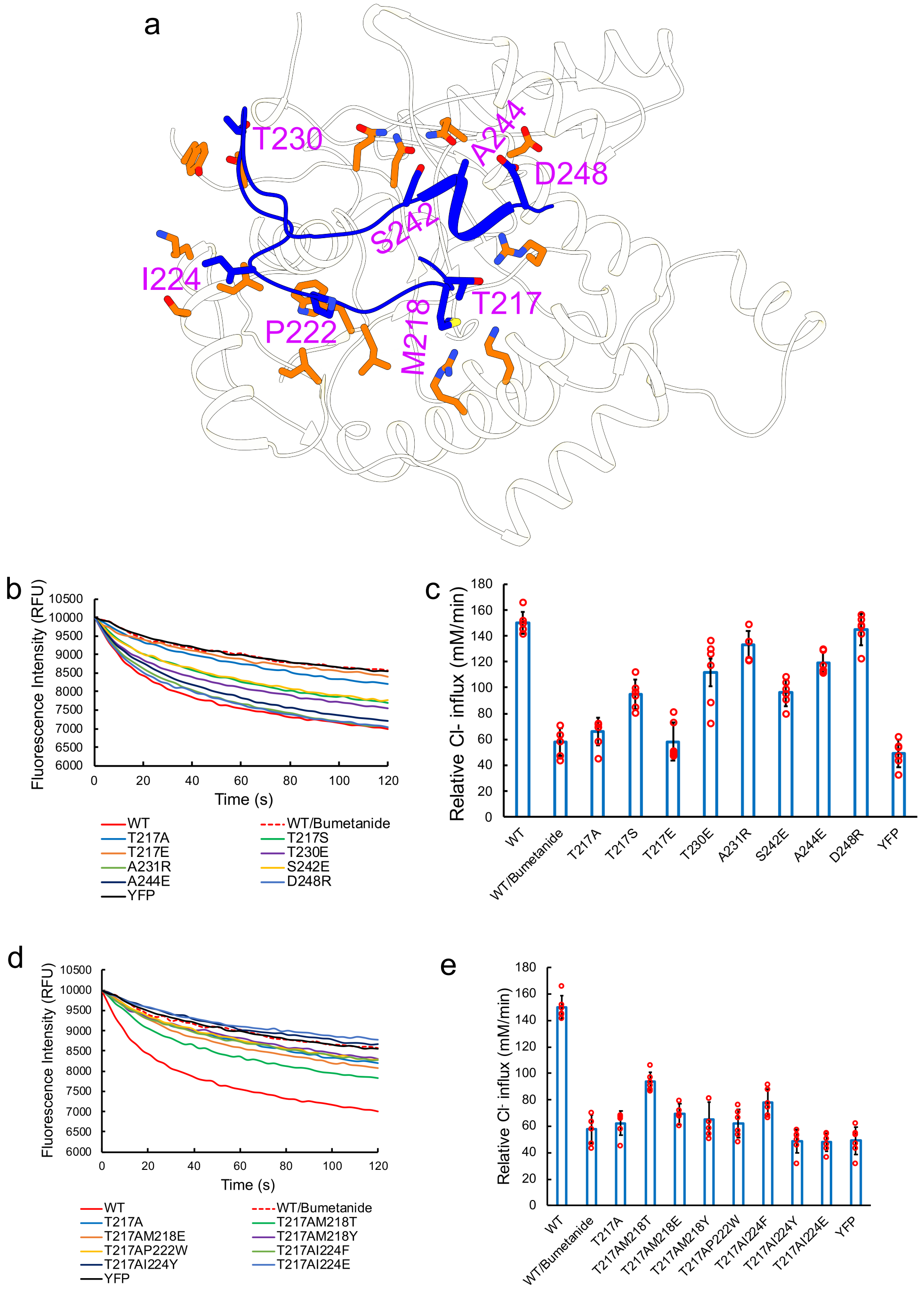

Supplementary Fig. 18 Functional analyses of phospho-mimic and other mutations designed to increase association between the N- and C-domains. a) NKCC1 structure highlights mutants examined in Cl^- influx assay in insect cells. (b-c) Mutations, including phosphomimic, do not lead to gain-of-function phenotype in our assay. (d-e) No second mutations were identified to rescue the loss-of-function phenotype of T217A. n=6 in all experiments; data are presented as mean values +/- SD.

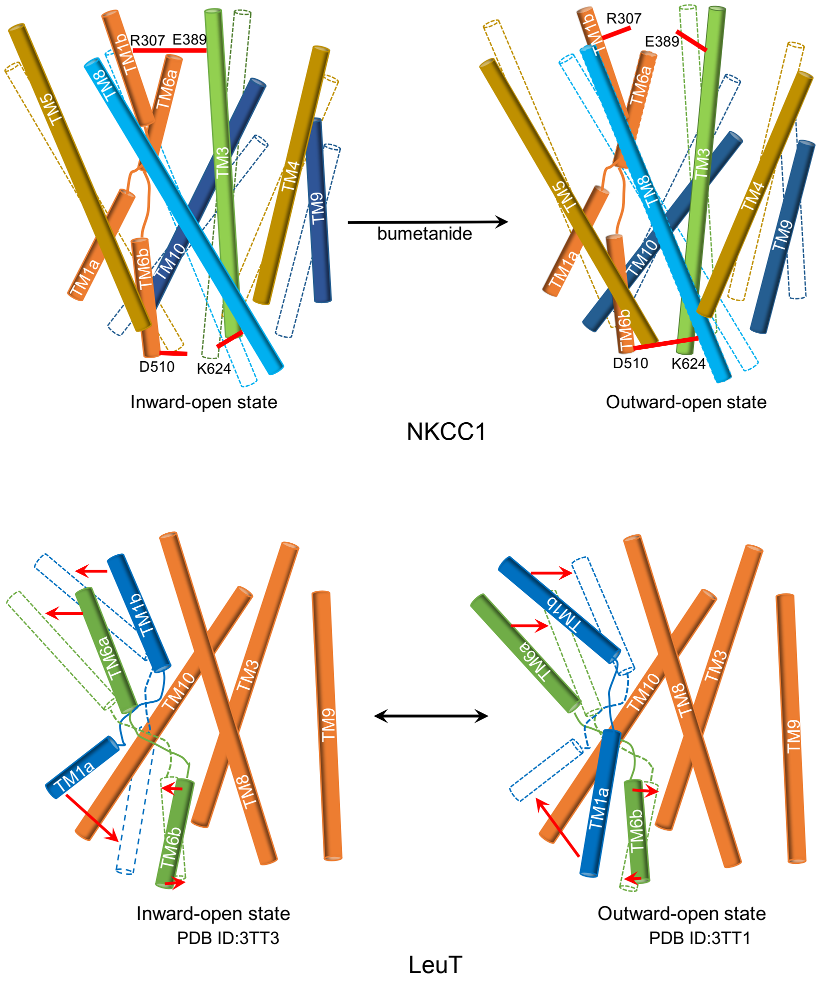

Supplementary Fig. 19 A gating model for NKCC1. (Top) Alternating between inward- and outward-open states involves reciprocal formation and breakage of salt bridge interactions at the extracellular and intracellular gates and movements of TM helices. In the inward-open state, the intracellular gate is open as the gating interaction (K624-D510) is broken, while the extracellular gate is closed by the salt bridge R307-E389. Bumetanide binding breaks the extracellular gate and triggers a concerted movement of TM3, TM4, TM5, TM8, TM9, TM10, and the ICL1 helix, fostering gating interactions that close the intracellular vestibule. (Bottom) Substrate transport by LeuT necessitates drastic hinge-bending motions of TM1 and TM6 helices around the discontinuous regions.

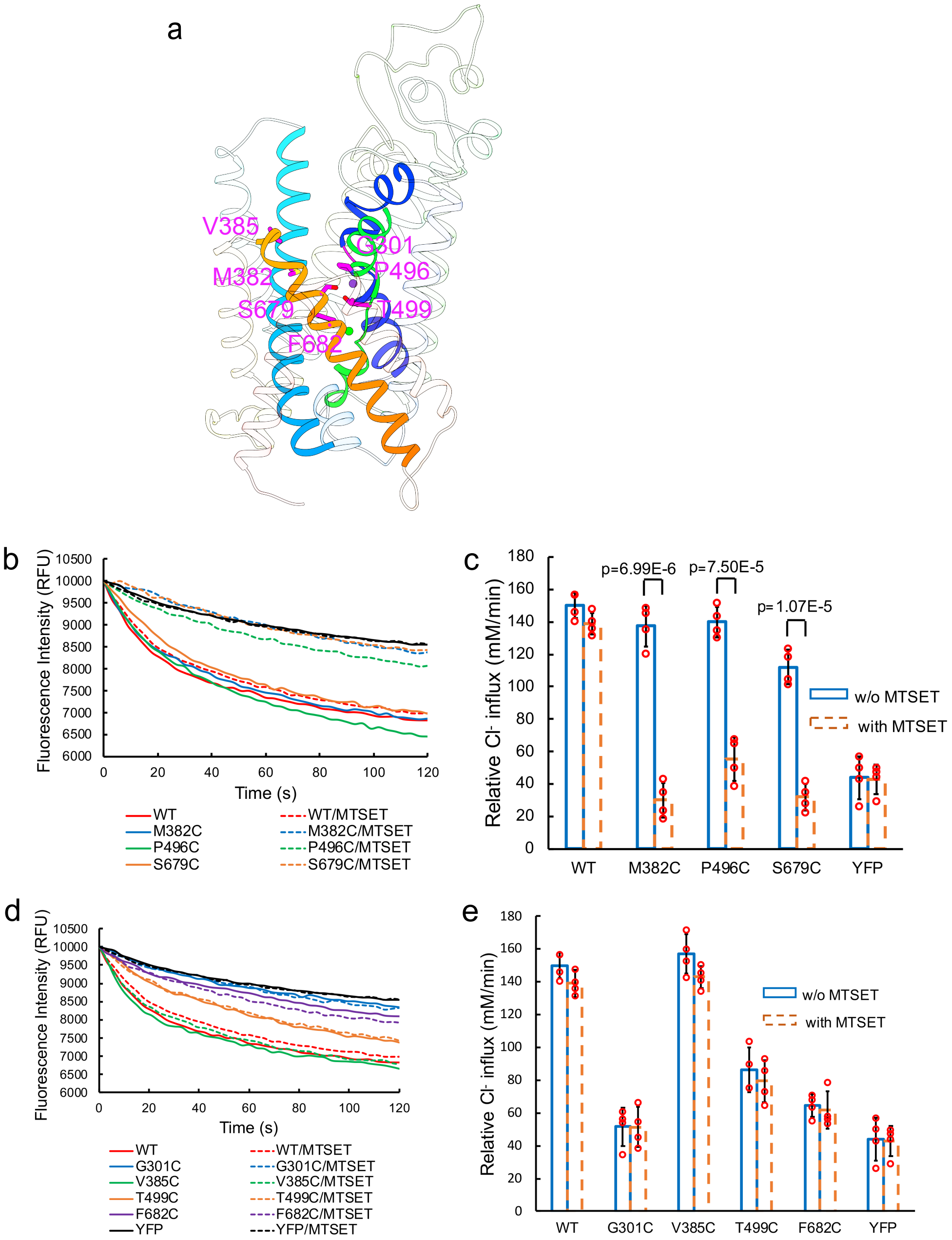

Supplementary Fig. 20 Analyses of the extracellular ion translocation pathway in NKCC1 by MTSET accessibility of substituted cysteines. a) Structure highlights cysteine substitution mutants examined in MTSET accessibility assays. b-c) M382C, P406C, and S679C are inhibited by MTSET. d-e) G301C, T499C, and F682C exhibit significantly decreased activity. V385C is not inhibited by MTSET possibly because its side chain does not directly point toward the extracellular vestibule. Unpaired one-tailed Student's t-tests are used for statistical analyses (n=4 in all experiments; data are presented as mean values +/- SD).

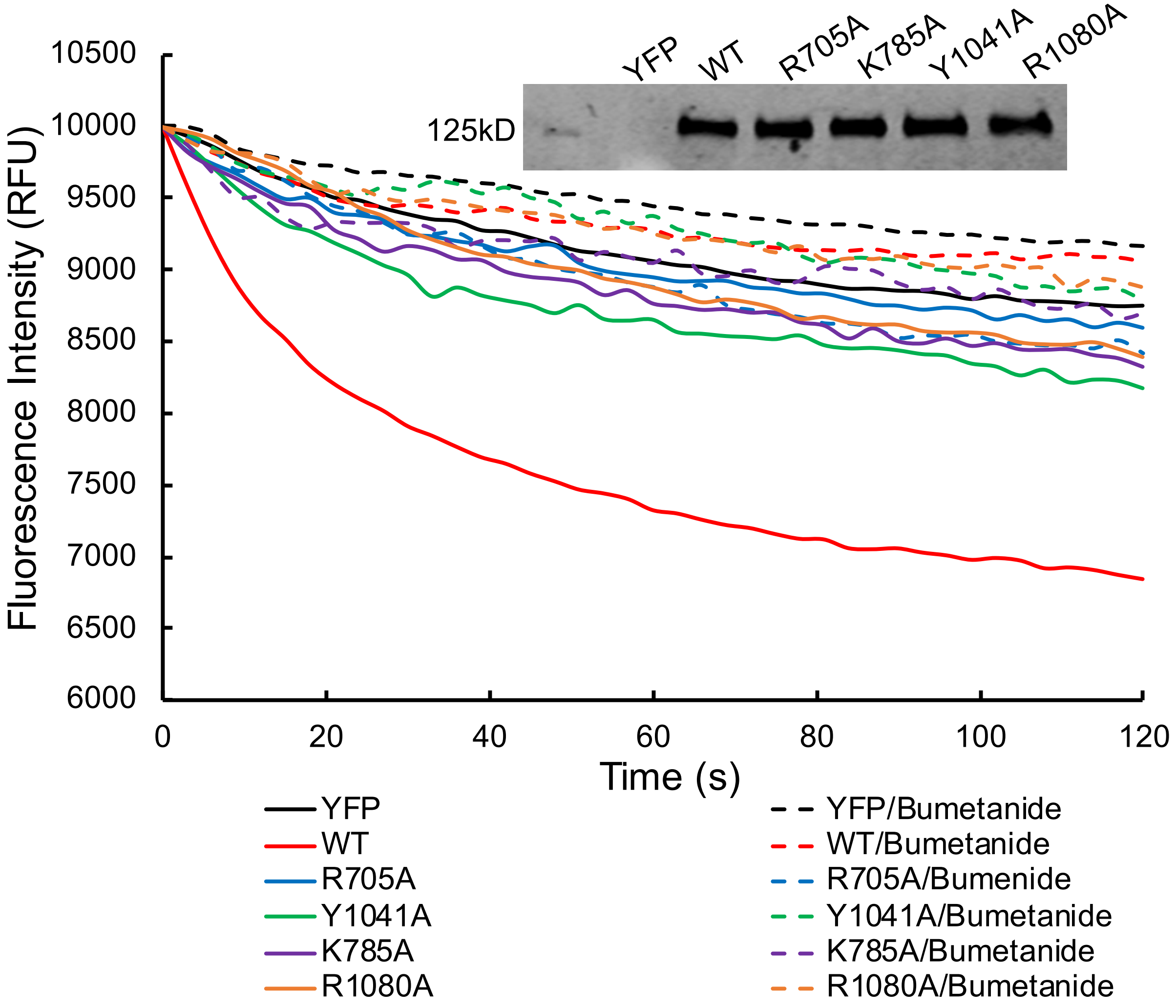

Supplementary Fig. 21 Functional analyses of NKCC1 mutants that affect association between the transmembrane and C-terminal domains. NKCC1 mutants show reduced transport activity due to disrupted association between the transmembrane and C-terminal domains. All mutants show normal expression level as the wildtype NKCC1 transporter in western blot assays.

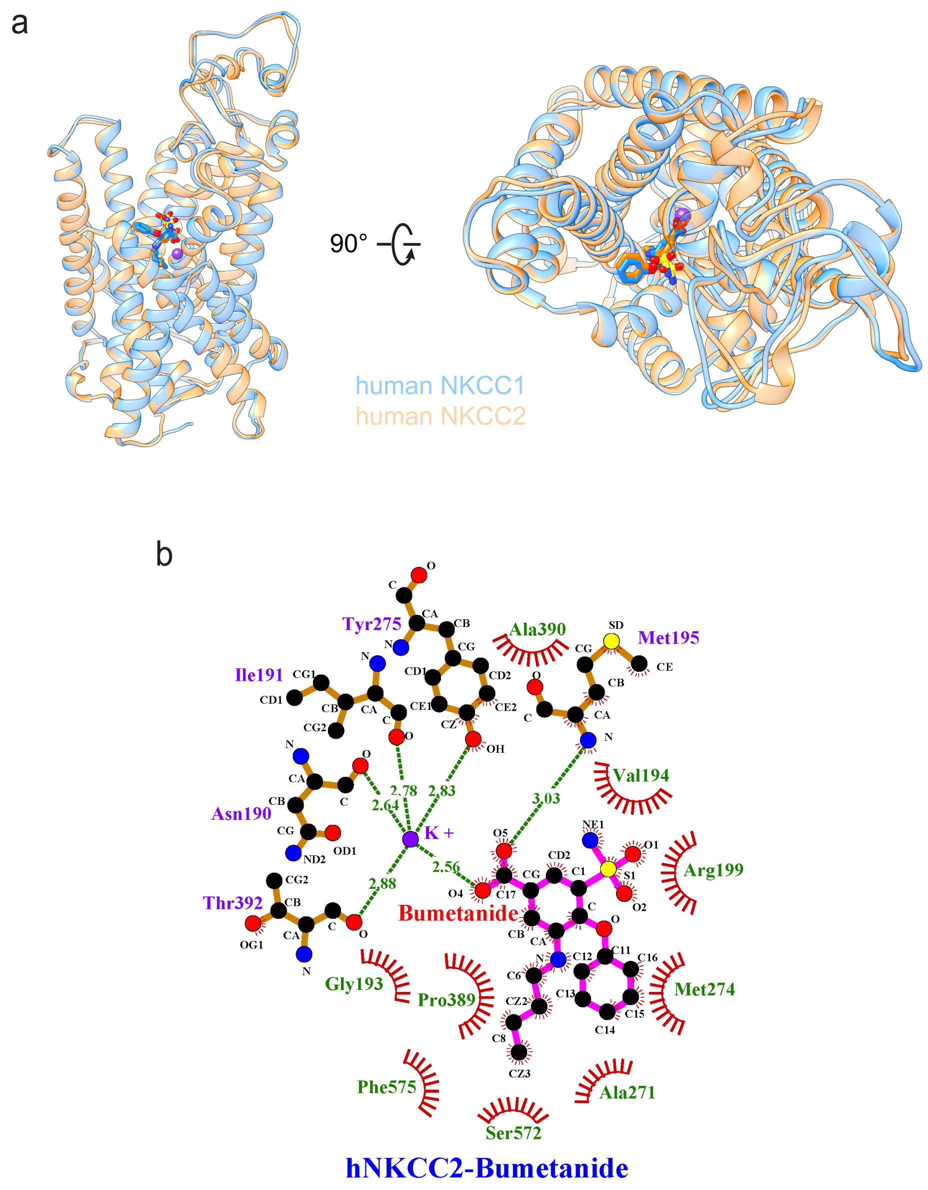

Supplementary Fig. 22 NKCC1 and NKCC2 harbor an almost identical bumetanide binding pocket. a) Experimental NKCC1 and predicted NKCC2 structures are almost superimposable. The bumetanide pose seen in the experimental NKCC1 map also closely matches the pose of docked bumetanide in the predicted NKCC2 structure. b) A 2D representation of bumetanide interactions with residues along the extracellular ion permeation path of the predicted NKCC2 structure. Each eyelash represents a hydrophobic interaction.

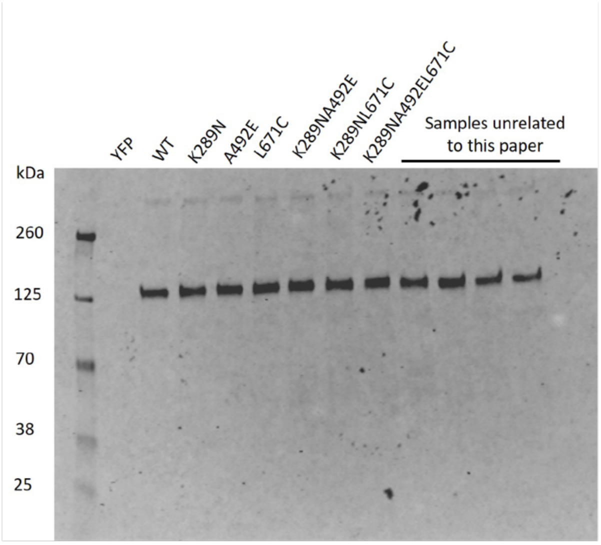

Supplementary Fig.2 Full Western Blot Gel

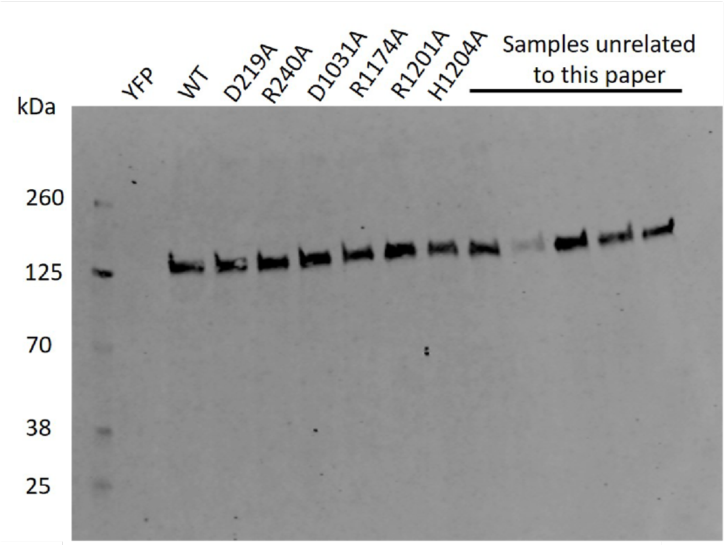

Supplementary Fig.16 Full Western Blot Gel

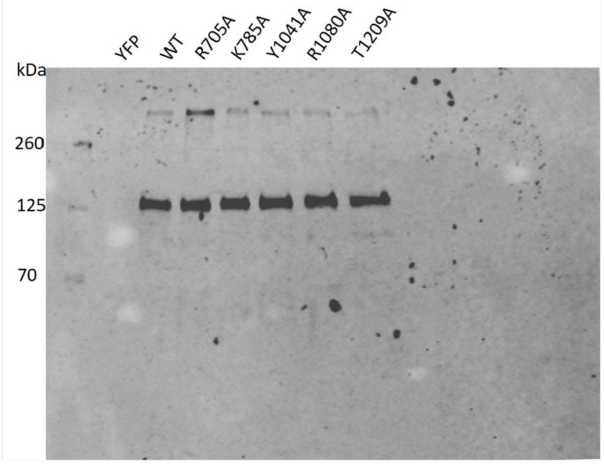

Supplementary Fig.21 Full Western Blot Gel
