# Supplementary material for: Structural basis for inhibition of the Cation-chloride cotransporter NKCC1 by the diuretic drug bumetanide
Source: Nat Commun. 2022 May 18;13:2747. doi: 10.1038/s41467-022-30407-3 (PMC9117670; doi:10.1038/s41467-022-30407-3)
Supplement: Supplementary file 3 — Description of Additional Supplementary Files [file 41467_2022_30407_MOESM3_ESM.pdf]

**File name: Supplementary Movie 1**

**Description: NKCC1 conformational changes.** Four views of a morph showing the transition between NKCC1 outward-facing and inward facing structures. The morph shows the transition from the outwardfacing structure of $NKCC_{iii}$ to the inward-facing structure (by homology to PDB-ID: 6NPL) and back outward. TM4,5 deep olive; TM1,6 orange; TM3 green; TM8 marine; TM9,10 blue; TM11,12 violet purple; ICL1 yellow; with gating residues R307, E389, D510, and K624 highlighted in red. $K^+$ and $Cl^-$ ions are colored purple and green, respectively.

**File name: Supplementary Movie 2**

**Description: LeuT NKCC1 comparison.** Comparing the transport conformational change in morphs of LeuT and NKCC1. The morphs show the transition from the outward-facing to inward-facing structures and back outward, for LeuT (PDB-IDs: 3TT3,3TT1) and NKCC1 (as in Movie 1). The “rocking bundle” consisting of TM1,2,6,7 is colored red; $K^+$ and $Cl^-$ ions are colored purple and green respectively.
